# Chrono-EEG dynamics influencing hand gesture decoding: a 10-hour study

**DOI:** 10.1038/s41598-024-70609-x

**Published:** 2024-08-30

**Authors:** Johanna Egger, Kyriaki Kostoglou, Gernot R. Müller-Putz

**Affiliations:** 1 Institute of Neural Engineering, Graz University of Technology, Graz, Austria; 2 BioTechMed, Graz, Austria

**Keywords:** Temporal variations, Gesture motor decoding, EEG, Movement-related cortical potential, Source space, Biomedical engineering, Data processing, Brain-machine interface

## Abstract

Long-term electroencephalography (EEG) recordings have primarily been used to study resting-state fluctuations. These recordings provide valuable insights into various phenomena such as sleep stages, cognitive processes, and neurological disorders. However, this study explores a new angle, focusing for the first time on the evolving nature of EEG dynamics over time within the context of movement. Twenty-two healthy individuals were measured six times from 2 p.m. to 12 a.m. with intervals of 2 h while performing four right-hand gestures. Analysis of movement-related cortical potentials (MRCPs) revealed a reduction in amplitude for the motor and post-motor potential during later hours of the day. Evaluation in source space displayed an increase in the activity of M1 of the contralateral hemisphere and the SMA of both hemispheres until 8 p.m. followed by a decline until midnight. Furthermore, we investigated how changes over time in MRCP dynamics affect the ability to decode motor information. This was achieved by developing classification schemes to assess performance across different scenarios. The observed variations in classification accuracies over time strongly indicate the need for adaptive decoders. Such adaptive decoders would be instrumental in delivering robust results, essential for the practical application of BCIs during day and nighttime usage.

## Introduction

A brain–computer interface (BCI) employs a non-muscular output pathway by exploitation of bioelectrical signals generated by the brain and modulated by the user’s intention to control technology or engage in communication with the external world^1,2^. People with neurodegenerative disorders, e.g., amyotrophic lateral sclerosis (ALS), trauma or stroke are at jeopardy to suffer from being completely paralyzed. In the case of ALS^3^, patients fear further progression of the disease into locked-in state (LIS)^4^ leaving them without a communication channel, besides the usage of eye movements and blinks to control augmentative and alternative technology^5^, while maintaining complete cognitive awareness. BCI technology can provide a remedy for such distressing conditions and improve quality of life by facilitating interaction with the outside world^6–9^.

The goal of the EU project INTRECOM is the development of such a BCI system for LIS patients unable to communicate orally. Through real-time motor and speech decoding using implantable electrodes positioned on the motor cortex of the hand and face area, communication via an external device shall be realized. Discrete and continuous cursor control will be implemented by decoding attempted hand movements of the paralyzed patient. Providing LIS patients with a means of communication in their home-environment will restore a degree of autonomy and independence^10^ enabling patients to call for their caregiver whenever necessary, e.g., at nighttimes during which caregivers cannot be constantly attentive regarding the needs of their patient. Thus, a crucial requirement for BCI systems is robust decoding of brain signals at every day- and nighttime^11^, hence stability of the recorded neural signals is vital as communication ability and autonomy of users heavily depend on it.

One prerequisite, however, for robust decoding are stable brain dynamics across time, a condition that does not typically occur in reality. The human electroencephalogram (EEG) has demonstrated diurnal variations in the delta^12,13^, theta, alpha and beta^14,15^ frequency bands during states of relaxed wakefulness in both closed and open-eye conditions. Typically, EEG exhibits lower power in the early morning and higher power during the afternoon and evening. Additionally, the peak of maximum power for lower frequencies occurs in the morning or afternoon, while higher frequencies tend to peak later in the day, suggesting an EEG frequency dependence associated with the time of the day^12,13^. Cacot et al.^12^ also observed a relationship between the diurnal changes in power across frequency components and recording conditions, such as whether the eyes were closed or open, as well as scalp topography.

These findings strongly suggest that the reported temporal variations throughout the course of a day may also appear in EEG dynamics during movement tasks. However, to date, there has been a lack of research aimed at understanding these effects as well as their implications on EEG movement decoding. We hypothesize that motor decoding performance could be substantially affected by these temporal changes, potentially influencing the efficacy and the reliability of BCI systems operating continuously throughout the day. This would highlight the necessity for the development of adaptive decoding in the context of BCI technology, especially for applications aimed at facilitating communication in home environments around the clock.

This research serves as preliminary work towards the objectives of the INTRECOM project. One of the aims of INTRECOM is to achieve stable and robust four-direction discrete cursor control, enhancing the user's degree of freedom and creating a more intuitive BCI system compared to a simple brain click^16^. Our objective is to contribute to the project goals by initially investigating the impact of temporal EEG variations on motor decoding performance, particularly focusing on movement-related cortical potentials (MRCPs). MRCPs^17,18^ are elicited during voluntary motor tasks (like movement execution, imagination, or attempts), and are usually revealed by locking the low-frequency EEG to the movement onset. They are known to encode various aspects of movement, including movement directions^19–22^, grasp types^23,24^, speed^25^ or force^26^. As part of this investigation, twenty-two young individuals were measured within a day with intervals of 2 h, while performing four right-hand gestures (corresponding to the four directions). MRCPs were analyzed across different sessions within the day. Additionally, we examined the spatial evolution of movement-triggered epochs in source space^27^.

## Methods

### Participants

Twenty-two healthy, right-handed participants (13 f/9 m) were recruited for the study. The average age of the study population was 26.4 ± 2.6 years as the inclusion criteria comprised a narrow age-group from 20 to 40 years to minimize the effect of changes in EEG power due to age^28^. Participants had a usual (work-) day routine of going to bed before 11 p.m. and waking up between 5 and 7 a.m. None of the included participants had worked night shifts for a year or traveled to a different time zone during the 2 weeks prior to participation. Volunteers consuming drugs or nicotine were excluded from the study. During the experiment, caffeine consumption was prohibited, hence participants stating to feel a physical or psychological effect of caffeine withdrawal after 24 h of caffeine absence were not considered in the study. The study was approved by the ethical review board of Graz University of Technology and conducted in accordance with the Declaration of Helsinki. Prior to the study, each participant gave a written informed consent. The study was conducted at the laboratory of the Institute of Neural Engineering of Graz University of Technology.

### Study procedure

**Fig. 1:**
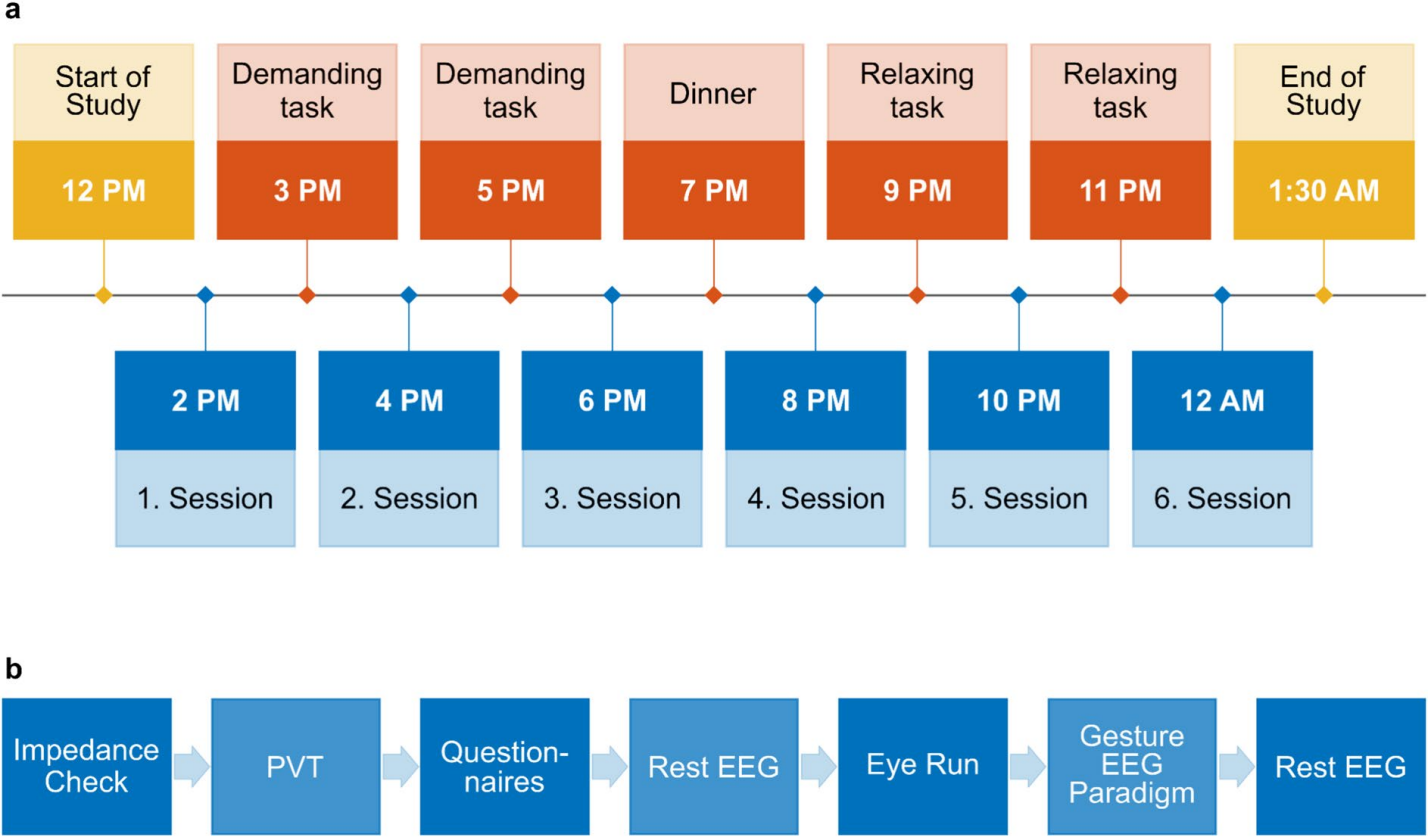
Study and session procedure. (**a**) Study process containing six recording sessions performed every 2 h starting at 2 p.m. until 12 a.m. (midnight). During the breaks between sessions, participants were instructed with specific tasks designed to mimic a usual working day. At 7 p.m. the participants were served a standardized dinner. The total duration of the study was 13.5 h. (**b**) Session procedure composed of seven blocks including an initial impedance check, a PVT, three questionnaires, a 2-min resting EEG measurement, a 6-min eye run for later eye artifact subtraction, the gesture EEG paradigm and another 2-min resting EEG measurement. The session lasted approximately 1 h.

The study procedure was designed in such a way as to mimic a usual working day including demanding tasks after the first and second measurement sessions. These tasks required active engagement from participants in both geometric and linguistic games. After the third recording session, dinner was served that was standardized for all participants and consisted of a pizza of choice. During the last two breaks between sessions, participants were given tasks to induce some sense of tiredness, as encountered in real-life work scenarios. These tasks involved watching a documentary as well as listening to music. Figure 1a shows the timetable of a study day that started at 12 p.m. and finished at 1.30 a.m. after midnight. The experiment consisted of six consecutive measurement sessions performed every 2 h at 2 p.m., 4 p.m., 6 p.m., 8 p.m., 10 p.m. and 12 a.m. (midnight).

Each session lasted approximately 1 h and was composed of several tasks (see Fig. 1b): (i) the EEG electrode check (to visually inspect the alignment of the cap) and impedance check, (ii) a psychomotor vigilance task (PVT)^29^, (iii) three questionnaires targeting emotions using the *Positive and Negative Affect Schedule *(*PANAS*)^30^, (iv) hunger with the *Hunger Level Scale* developed by the NEMO (Nutrition Education Materials Online) Management Group of Queensland Government, (v) tiredness by making use of the *Tiredness Symptoms Scale* (*TSS*)^31^, (vi) a 2 min resting EEG recording, (vii) one 6 min eye run to simultaneously record the participant’s EEG and electrooculography (EOG) while performing blinks and eye movements for later eye artifact attenuation (viii) the experimental gesture EEG paradigm as described below and (ix) another 2 min resting EEG recording.

In total, 20 complete sets of six measurement sessions were obtained. Due to technical incidents, one measurement session had to be discarded for two participants.

### Gesture EEG paradigm

**Fig. 2:**
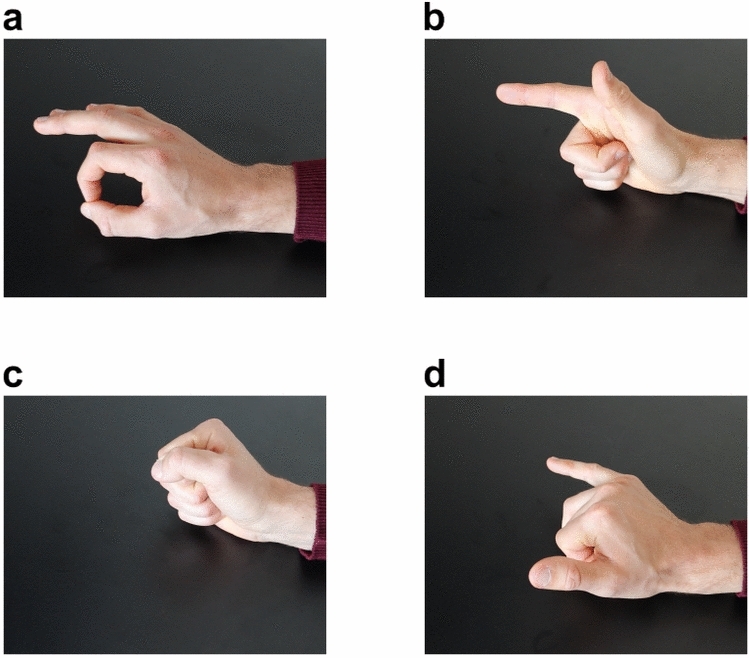
Gestures performed by the participant during the EEG recording of the gesture paradigm. The following gestures are depicted: (**a**) pincer grasp, (**b**) pistol, (**c**) fist, and (**d**) “Y”-gesture of the American sign language. During the paradigm, execution of the gestures was indicated by a visual cue presented on a screen. The participants were instructed to hold the indicated gesture for 3 s until the appearance of a blank screen.

**Fig. 3:**
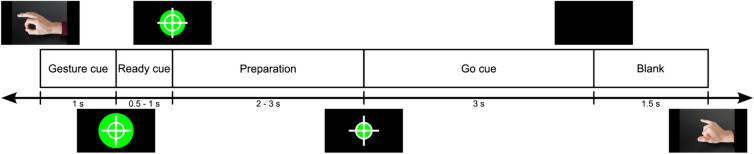
Duration and timing of one trial of the gesture EEG paradigm. The gesture to be executed was indicated by a photograph at the beginning of a trial. After 1 s, the “Gesture cue” disappeared and a filled green circle in addition to a white inner circle and a fixation cross representing the “Ready cue” took its place for a random time interval of 0.5–1 s. During the preparation period, the filled green circle in the background shrunk at random speed for 2–3 s. At the time of the “Go cue”, the filled green circle had hit the inner white circle indicating the start of movement execution. The position of the gesture was kept for 3 s after which a blank screen appeared signaling the end of a trial. One trial duration varied randomly between 8 and 9.5 s.

The study investigated four different right-hand gestures. The selection of the number of classes was made with the intention of later assessing the efficacy of a classifier designed for a four-direction decoder as part of the INTRECOM project. Each participant was comfortably seated in front of a computer screen positioned at a distance of 50–60 cm while placing the right hand on a table inside a wooden box. The participant was requested to follow the instructions shown on the computer screen. In addition, the participant was asked to avoid eye blinking and swallowing during the duration of a trial period. The gesture EEG paradigm was designed based on work of Ofner et al.^32^. At the beginning of each trial, the class cue was presented for 1 s corresponding to one of four classes: fist, pistol, pincer grasp and Y-gesture of the American sign language (see Fig. 2). The detailed timing of one trial can be seen in Fig. 3. Here, a fixation cross in front of a filled green circle with a smaller inner white circle was displayed after the cue for a random time interval of 0.5–1 s. The participant was asked to focus their gaze firmly on the cross to avoid eye movements. The presentation of the fixation cross was followed by the preparation period of 2–3 s in which the green circle started to shrink with random speed towards the size of the inner white circle. When the filled green circle touched the inner white circle, the participant was instructed to perform the gesture corresponding to the previously shown class cue. The participant was then asked to hold the indicated gesture for approximately 3 s until the screen went black indicating the end of a trial. Between trials, a break of 1.5 s allowed the participant to go back to the initial position and prepare for the next trial. The total duration of a trial period varied between 8 and 9.5 s.

The gesture EEG paradigm consisted of eight movement runs of approximately 5 min with 30 s of break in between. Each run was composed of 32 trials (random cues, balanced), resulting in 256 recorded trials per session and per participant. During the 30 s breaks the participants were instructed to fill out a visual analogue scale (VAS) to assess their level of fatigue.

During each session and before the gesture EEG paradigm, an *eye run* was performed to simultaneously collect the participant’s EEG and EOG activity while at rest with eyes open, capturing eye movements such as blinks, vertical and horizontal eye movements^33^. The collected data was then used to train an eye artifact attenuation algorithm as described by Kobler et al.^33,34^.

### Questionnaires

During each of the sessions prior to the gesture EEG paradigm, participants were asked to complete questionnaires evaluating their state in terms of emotion, hunger, tiredness, and fatigue. These questionnaires were either given in English or translated to German depending on the participant’s preferences. To assess the participant’s emotions, the *Positive and Negative Affect Schedule* (*PANAS*)^30^ was used, consisting of twenty different emotional states (e.g., interested, excited, upset) rated on a scale from 1 to 5 (1 being low and 5 being high). The PANAS score was divided into a positive and negative affect score by calculating the average of the ten positive and ten negative emotions, respectively. A non-standardized questionnaire introduced by the NEMO Management Group of Queensland Government was used to evaluate the participants' level of hunger. The questionnaire listed several physical sensations of hunger, and participants were asked to pick the most suitable one on a scale from 1 (indicating starvation) to 10 (indicating a full, uncomfortable feeling in the stomach). Furthermore, various symptoms of tiredness were evaluated using the *Tiredness Symptoms Scale *(*TSS*)^31^ listing fourteen symptoms rated from 0 to 14 (with 0 indicating no impact and 14 indicating a significant impact). In addition, during each of the seven 30 s breaks between paradigm runs, the participants were instructed to rate their level of fatigue using a visual analogue scale (VAS) in the range of 0 (not at all fatigued) to 10 (extremely fatigued).

### Recording

For the EEG measurements, 60 active, gel-based electrodes (actiCAP Brain Products GmbH, Germany) were placed on the scalp according to the 10–10 international electrode standard setup covering frontal, central, parietal, occipital and temporal areas. Four additional active electrodes were used to measure the electrical activity induced by the dipolar behavior of the eyes and were positioned at the outer canthi of the eyes as well as on the inferior and superior of the left eye. The reference and ground electrode were placed on the right mastoid and the forehead at the position of FPz, respectively. The EEG and EOG signals were recorded at 500 Hz using biosignal amplifiers (BrainAmp, Brain Products GmbH, Germany).

A video camera was placed above the participant’s right hand inside a box, with a green marker attached to the nail of the right index finger to enable movement tracking by a motion capture system developed at the institute. The sampling frequency of the camera was 30 Hz.

The EEG cap was worn continuously throughout the entire study without being removed. Since we opted for a gel-based setup, cap shifting due to head movements was minimized. Participants were instructed to avoid sudden movements or scratching their heads throughout the study. In the case of an itchy scalp, participants were permitted to use the blunt needles used for gel application in a gentle and cautious manner for relief.

### Preprocessing

The recorded signals were processed using MATLAB R2022b (MathWorks. Massachusetts, USA) and the open source toolbox EEGLAB^35^. At first, the raw EEG signals were inspected visually, channels contaminated by noise were identified and interpolated by the four closest electrodes weighted by their inverse distance to the bad channel. Further, the 50 Hz line noise and its first harmonic at 100 Hz were removed using a Butterworth bandstop filter of 2nd order. A Butterworth highpass filter of 5th order with a cutoff frequency of 0.3 Hz was employed to avoid stationary artifacts such as drifts. High frequency noise above 70 Hz was attenuated using a Butterworth lowpass filter (of order 70). In order to remove EOG artifacts such as blinks or saccades, an eye artifact attenuation model was trained on the pre-recorded and processed eye run data of each participant using the SGEYESUB algorithm, as described by Kobler et al.^34^. The most frontal row of electrodes (AF) was not included in following processing steps to minimize the impact of residual artifacts related to eye movements. Pops and drifts in the EEG signals were attenuated using the HEAR algorithm^36^. Subsequently, temporal electrodes (FT7 and FT8, T7 and T8, TP7 and TP8) were excluded due to their higher noise levels compared to the other channels and their lack of relevant information for subsequent analysis in both sensor and source-space.

### Movement-related cortical potentials

MRCPs were extracted using a lowpass Butterworth filter (4th order) with a cut-off frequency of 3 Hz. The data were segmented into epochs of 5.5 s, ranging from 2.5 s before to 3 s after movement onset. The movement onset was obtained from simultaneous recordings of the movement of the right index finger using a motion capture system that extracted the x- and y-coordinates of the green marker. By calculating the speed and applying thresholding, the onset of the movement was detected. In order to remove bad trials to reduce the impact of transient artifacts, trial rejection based on a threshold of ± 100 µV was performed on the broad-band data. These trials were then removed from the lowpass-filtered epochs. On average, approximately 7 of the original 256 recorded trials were removed per participant and session. Additionally, 15 trials had to be excluded from further analysis as the motion capture system was incapable of tracking the movement. After downsampling the epochs to 9 Hz, the data was re-referenced to a common average reference.

### Analysis of MRCPs

In order to examine temporal variations in the dynamics of MRCPs, we first assessed whether MRCPs associated with a particular gesture exhibited significantly greater deviations over time compared to those associated with other gestures. Therefore, we averaged epochs of a session and a specific gesture to obtain the MRCPs at each channel for every participant. The negative peak of the motor potential and the positive peak of the post-motor potential were then extracted. Their relative values were calculated by comparing them to those obtained during the initial measurement session at 2 p.m. In order to investigate variations among gestures and across different sessions, a Kruskal–Wallis test^37^ was conducted with a statistical significance level of 5%. To address the issue of multiple comparisons, p-values were adjusted following the Benjamini–Hochberg procedure^38^.

#### Amplitude of motor and post-motor potential

To examine potential deviations in MRCPs over time regardless of the executed gesture, the amplitudes of both the motor and post-motor potentials were isolated from the average MRCPs for each channel across all four gestures for each measurement session. A Wilcoxon signed rank test^39^ was then employed for every channel to assess statistical significance between every pair of sessions, using a significance level of p = 0.05. As before, the method of Benjamini–Hochberg^38^ was applied to correct the p-values for multiple comparisons regarding the number of channels and sessions.

To explore the correlation between fatigue, tiredness, and the amplitudes of the MRCPs, we utilized linear mixed-effect (LME) models. LME models represent statistical models that are useful in the context of studies with repeated measurements to account for correlations between multiple observations. These models incorporate fixed effects, which are the ones of interest, and random effects that account for variations between subjects or experimental conditions^40^. Here, we implemented LME models with participant-dependent intercepts to investigate the influence of fatigue and tiredness on MRCP amplitudes. Specifically, we employed univariate LME models for each channel, using VAS fatigue assessments (averaged across the seven results obtained during the breaks between measurement runs of the gesture EEG paradigm) or TSS scores as independent variables. The response variables in these models were the amplitudes of either the motor or post-motor potentials within each channel. The LME p-values for each channel and analysis were corrected in terms of multiple comparisons by using the procedure of Benjamini–Hochberg^38^.

#### Temporal evolution of MRCP dynamics

For further examination of temporal changes in MRCP dynamics, a Wilcoxon rank sum test^39^ was employed to compare MRCP patterns between every pair of sessions. Therefore, epochs of all four gestures and all participants were combined and statistical testing was performed for each channel and at each timepoint of the movement epoch. A sampling frequency of 9 Hz was used. To control for the false discovery rate introduced by multiple testing of sessions, channels and timepoints, as previously, Benjamini–Hochberg procedure^38^ was implemented.

#### Source localization

To obtain the brain sources and induced power changes associated with movement, we made use of the Brainstorm toolbox provided by Tadel et al.^41^. We computed the head models by employing boundary element methods in accordance with the MNI/Colin27 anatomy template included in Brainstorm and the general EEG electrode positions embedded in EEGLAB. For the estimation of the noise covariance matrix, we estimated the average covariance of 1 s intervals from the first preprocessed resting EEG recording of each session across conditions. As input, we used epochs within the time interval from − 2.5 to 3 s around the movement onset and within the frequency range of 0.3 to 70 Hz sampled at 500 Hz. These epochs were averaged across conditions and participants. For in-depth analysis of specific frequency bands, we bandpass filtered the signals with a stopband attenuation of 60 dB in the frequency range of delta (0.5–3.5 Hz), theta (4–7.5 Hz), alpha (8–12.5 Hz), beta (13–29.5 Hz) and gamma (30–70 Hz) using Brainstorm. Subsequently, we used the standardized low-resolution brain electromagnetic tomography (sLORETA) to estimate the 15,002 brain sources with dipole orientations perpendicular to the cortical surface^27^. As described in Pereira et al.^19^, we retrieved the significantly different voxels with respect to a baseline, from − 1.75 to 1.5 s relative to the movement onset, by implementing a parametric *t*-test. To address the issue of multiple comparisons, Benjamini–Hochberg correction^38^ was applied for the number of voxels, timepoints, measurement sessions and frequency bands to adjust the p-values.

### Classification

For the classification of the four gestures, a multiclass shrinkage linear discriminant analysis (sLDA)^42,43^ classifier was applied to the extracted epochs. As input, causal windows of 1 s were shifted along the epochs in steps of 1/9th of a second. Feature extraction windows of 1 s had previously yielded promising results in Ofner et al.^32^. Classification was performed offline and individually on each participant. To investigate whether variations in EEG during movement tasks over time influence the decoding capability of a classifier, five decoding strategies were employed.

To assess the general decoding capability of a classifier being trained on data collected at various timepoints throughout the day and night, two approaches were investigated. Strategy 1 made use of a fivefold cross-validation by combining four folds of each session as a training set. The trained classifier was then applied to the remaining fold of each measurement session. Strategy 2 was based on a leave-one-out approach by using five sessions as training set and the remaining session as testing set. This strategy was applied to each one of the six sessions by training on all sessions except for the corresponding one used as a test set. Two of the participants did not have a complete set of measurements, therefore only twenty participants were considered for this approach.

To assess the performance of a classifier in the context of continuous data inflow, three training schemes were employed. Strategy 3 considered a fivefold cross-validation within each session individually. Strategy 4 used an incremental number of sessions as training set and subsequently applied the model on the next measurement session in chronological order (i.e., train on session 1 and test on session 2, then train on session 1 and 2 and test on session 3 etc.). Strategy 5 combined the first two sessions as a training set and evaluated the model on the remaining four sessions. As stated previously, for the fourth and fifth training scheme two participants had to be excluded as their datasets were incomplete.

## Results

### Behavioral data

**Fig. 4:**
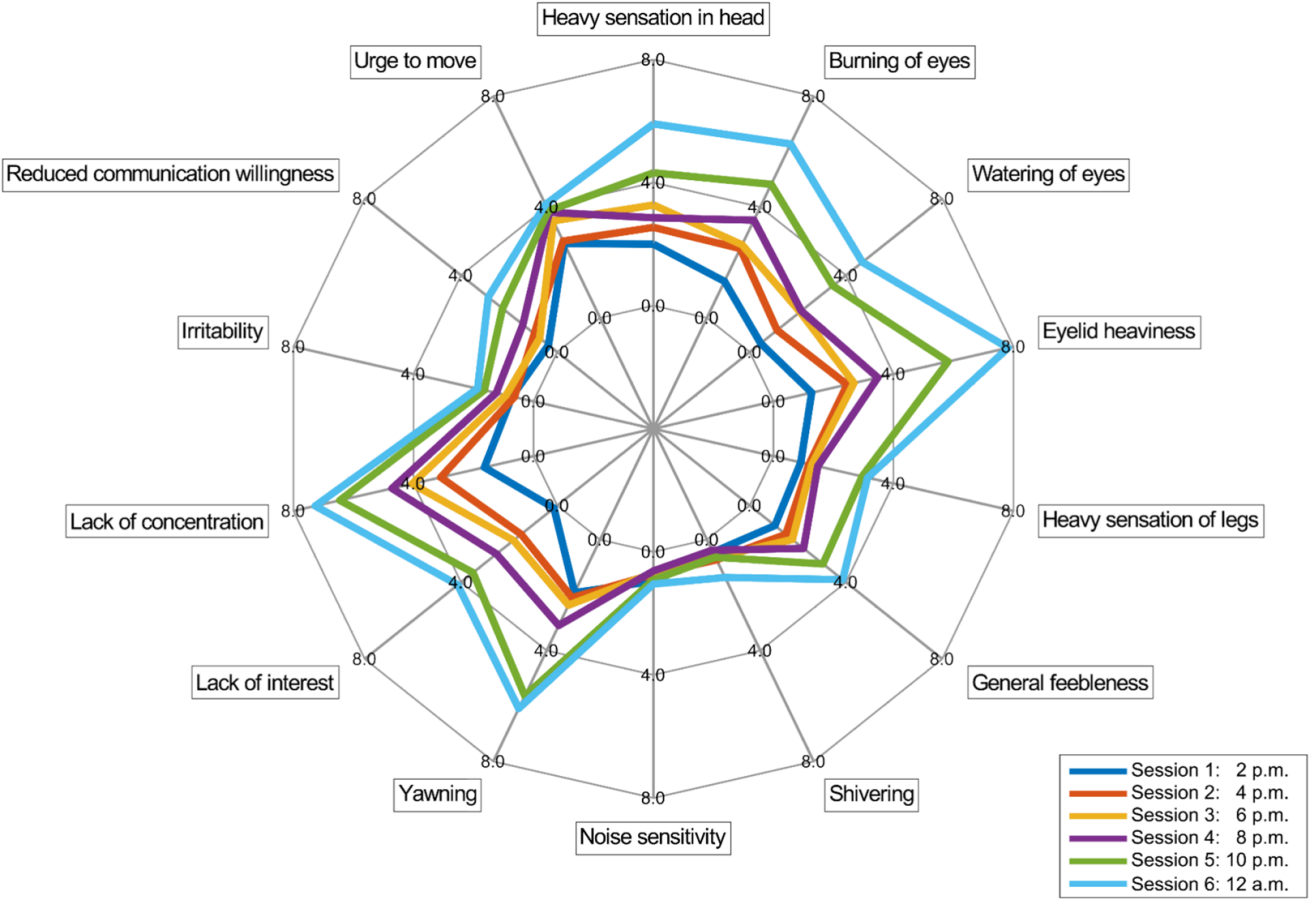
Evaluation of the TSS averaged across participants. The grand average ratings for each of the tiredness symptoms and measurement sessions are depicted as a spider chart. For visualization purposes, the assessment scale was abridged to a maximum rating of 8 instead of 14. An increase in rating for each symptom was observed from session 1 at 2 p.m. until session 6 at 12 a.m.

Analysis of the averaged TSS questionnaire responses across participants revealed, as depicted in Fig. 4, that the intensity of each tiredness symptom became more pronounced over the course of the study. The evaluation of the averaged responses indicated that symptoms including a sensation of heaviness in the head, burning and watering of the eyes, eyelid heaviness, yawning, lack of interest, and diminished concentration became more prominent as the study progressed. Tiredness symptoms including shivering, noise sensitivity, irritability, and the urge to move remained at an almost constant level throughout the duration of the study.

The evaluation of the questionnaires targeting emotions^30^ and hunger level (see Supplementary Fig. S4), as well as the analysis of the VAS (see Supplementary Fig. S3) filled out during breaks between runs of the gesture EEG paradigm can be found in the Supplementary Material. In Supplementary Fig. S3, we observed a gradual increase in the level of fatigue from one break to another within each session. Additionally, when comparing breaks of different sessions, the level of fatigue increased as the recordings took place later in the day. Participant hunger levels, as shown in Supplementary Fig. S4, initially rose until dinner at 7 p.m., subsequently decreasing post-dinner. However, as the experiment progressed, hunger levels increased again compared to post-dinner levels. When examining the evaluation of the PANAS^30^ in Supplementary Fig. S4, a gradual decline in positive emotions from the beginning until the end of the study was observed. The negative affect score presented a different picture, wherein negative emotions diminished until dinner, slightly increased after dinner and persisted until the end of the experiment.

### Analysis of MRCPs

The statistical test on MRCP amplitudes (motor and post-motor potential) corrected for multiple comparisons revealed no statistically significant differences between gestures (p > 0.05). We therefore concluded that a combination of trials independent of associated gesture is appropriate for subsequent analyses.

#### Amplitude of motor and post-motor potential

**Fig. 5:**
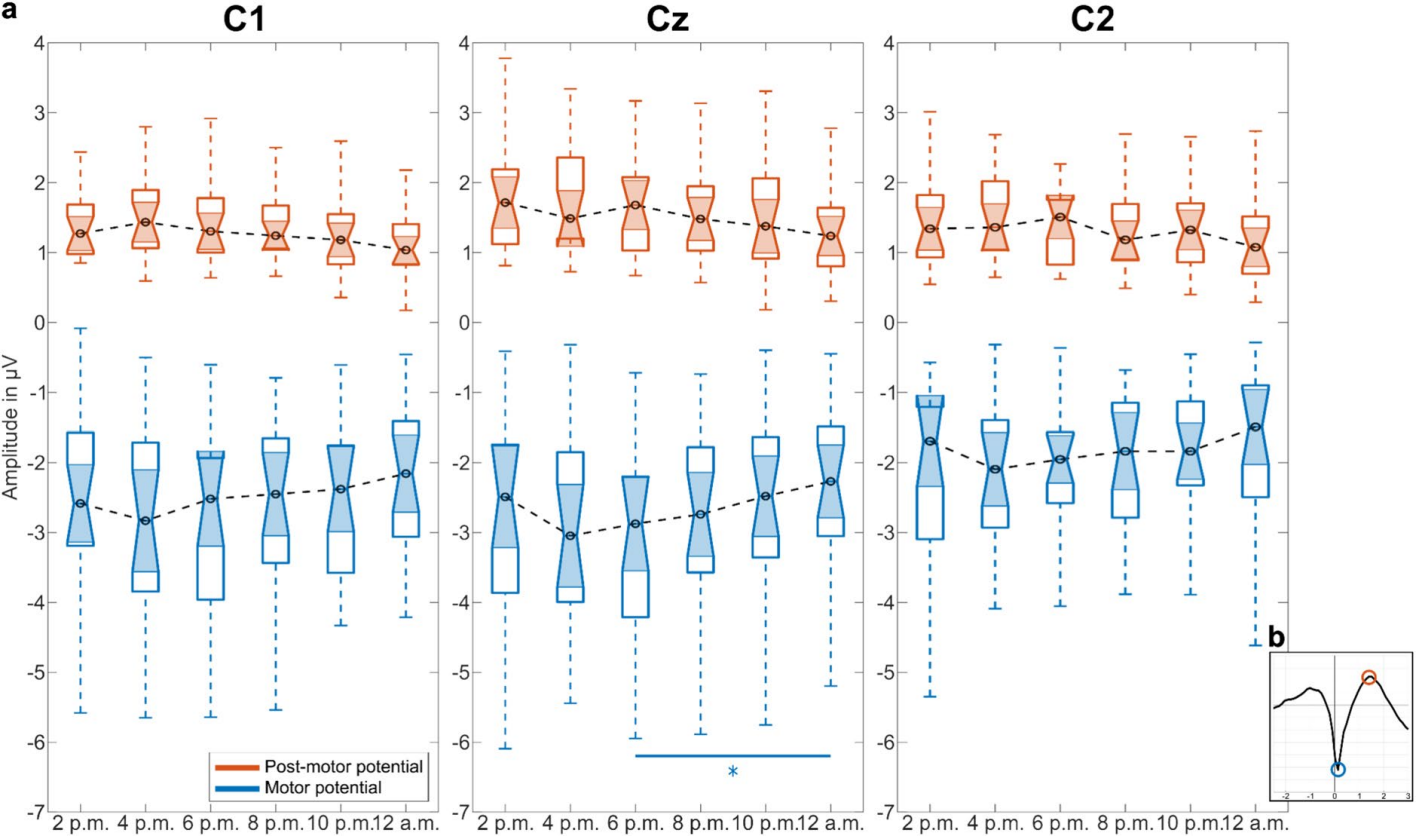
Temporal changes in amplitudes of the motor (blue) and post-motor potential (orange). (**a**) Electrode positions C1, Cz and C2 above the motor cortex are visualized. The box-whiskers-plots represent the distribution among participants including levels of statistical significance (*p < 0.05) in Cz between recordings at 6 p.m. and 12 a.m. determined by a Wilcoxon signed rank test. The median is depicted as black dashed line. In the motor potential, an increase in amplitude from 2 p.m. until 4 p.m. is followed by a gradual decrease until midnight. The evolution of the post-motor potential differs from electrode to electrode, however a decrease in amplitude from 2 p.m. until 12 a.m. is apparent. (**b**) Graphical explanation of the motor and post-motor potential amplitudes that were extracted from the MRCP waveform for each participant and session.

To analyze MRCPs in terms of motor and post-motor potential amplitudes, we concentrated on three electrodes symmetrically positioned above the sensorimotor areas, specifically C1, Cz and C2. The amplitude variations in motor and post-motor potentials among participants are illustrated over time in Fig. 5a. Motor and post-motor potentials, extracted as demonstrated in Fig. 5b, were notably more pronounced in electrodes C1 and Cz, located above the contralateral hemisphere corresponding to the side of the movement, compared to those recorded in C2. Across all three electrodes, a decrease in amplitude was observed from 6 p.m. until midnight for both motor and post-motor potential. The median of the motor potential decreased from 1.3 to 1.04 µV in C1, from 1.68 to 1.24 µV in Cz and from 1.51 to 1.08 µV in C2, whereas the median of the post-motor potential diminished from − 2.52 to − 2.16 µV in C1, from − 2.88 to − 2.27 µV in Cz and − 1.95 to − 1.49 µV in C2. The decline in motor potential amplitude commenced with a slight increase between 2 p.m. and 4 p.m. for the median of the amplitude in C1 (from 1.27 to 1.43 µV) and C2 (from 1.34 to 1.36 µV). Statistically significant differences (p = 0.0434) in motor potential amplitude at the central electrode Cz were evident in recordings made at 6 p.m. and 12 a.m.

**Fig. 6:**
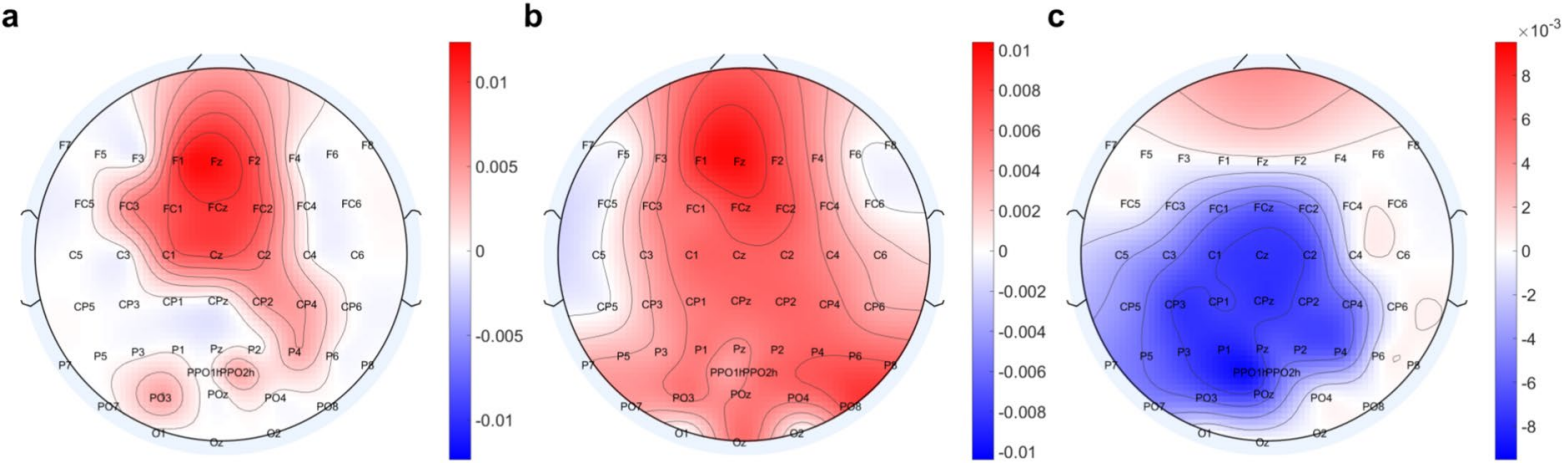
Topographical visualization of the statistically significant associations between (**a**) VAS scores and motor potential amplitude (p < 0.0498), (**b**) TSS scores and amplitude of the motor potential (p < 0.0496), and (**c**) TSS scores and the amplitude of the post-motor potential (p < 0.0488). Positive associations are depicted by red hues whereas negative associations are indicated by blue hues.

Regarding the relationship between MRCP amplitudes and fatigue/tiredness, in Fig. 6 we present topographical distributions of the estimated LME coefficients, depicting the relationship between averaged VAS scores, TSS scores, and the amplitudes of both motor and post-motor potentials. Only statistically significant associations are depicted. The results reveal a positive correlation between motor potential amplitude and subjective responses to the fatigue-related VAS scores (Fig. 6a) and TSS scores (Fig. 6b), with the central and slightly contralateral to the movement electrodes exhibiting a stronger relationship (Fig. 6a). This indicates that as fatigue levels and tiredness symptoms increase, the negative amplitude of the motor potential decreases in magnitude. Figure 6c presents only the significant estimated coefficients for the univariate LME models, which shows that increasing tiredness is associated with a reduction in amplitude of post-motor potentials in the contralateral centro-parietal and parietal regions relative to the dominant hand. In contrast, our analysis using VAS fatigue levels as an independent variable and post-motor potential amplitude as a dependent variable did not reveal any statistically significant associations (p > 0.05).

#### Temporal evolution of MRCP dynamics

**Fig. 7:**
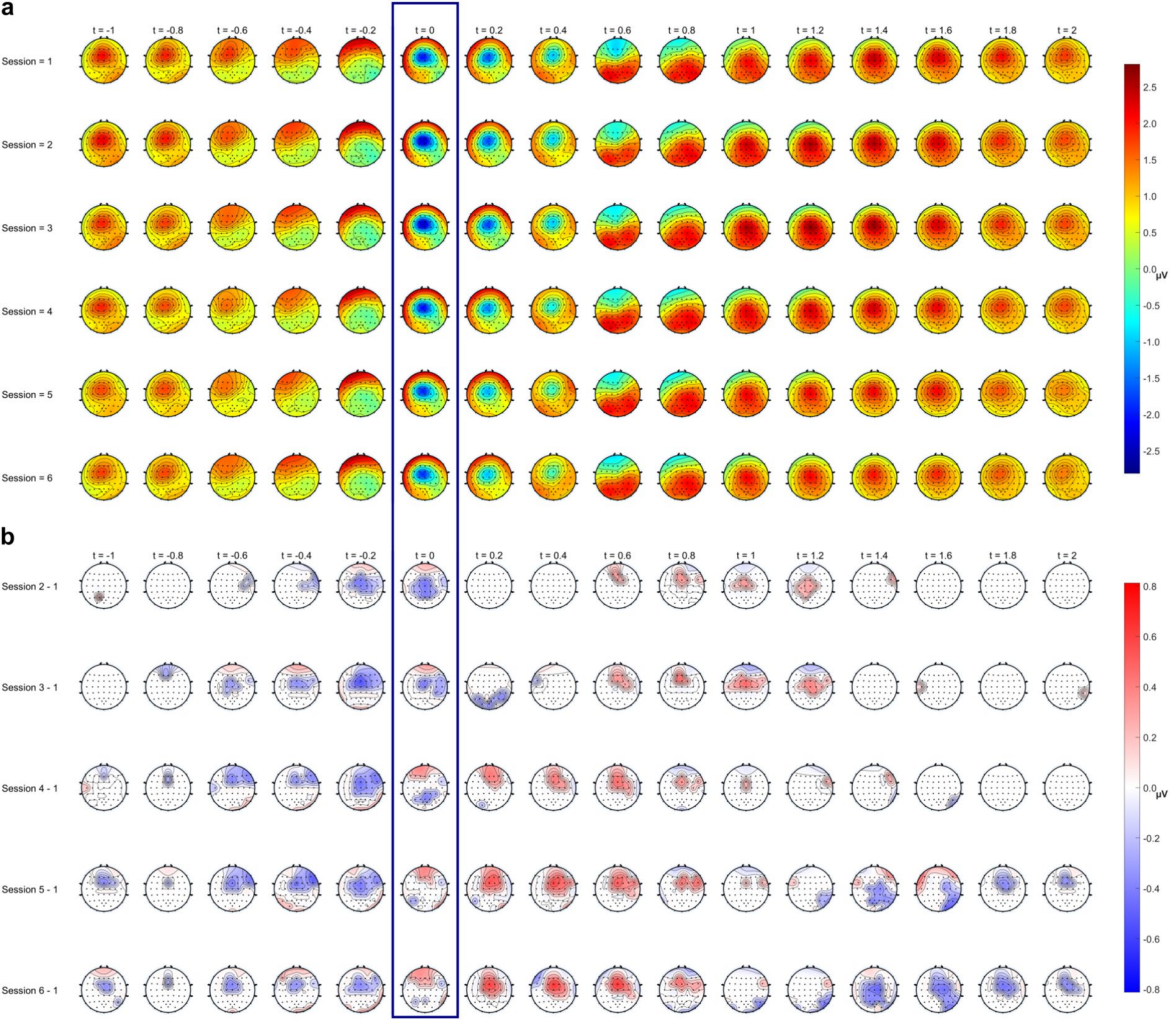
Temporal changes in MRCP dynamics visualized as topographical representations to demonstrate spatial distributions. Depicted are the grand average MRCPs for specific timepoints around the movement onset denoted at timepoint t = 0 s for (**a**) each session without statistical masking and (**b**) the difference between the corresponding session and session 1 (investigated session—session 1) with statistical masking (p < 0.0498).

To gain comprehensive insights into the temporal changes of MRCP dynamics and their spatial distribution, we illustrate in Fig. 7 the progression of MRCPs, averaged over all four gestures, in the form of topographical representations over several timepoints around the movement onset (indicated at t = 0 s). Figure 7a shows solely the magnitude of the MRCPs whereas Fig. 7b depicts the difference in MRCP amplitude values between the corresponding session and session 1 (investigated session—session 1) only for the areas that show statistically significant deviations (p < 0.0498) to session 1 corrected for multiple comparisons. As depicted in Fig. 7b, we found that variations in MRCPs became more pronounced with increasing time intervals between recordings.

#### Source localization

**Fig. 8:**
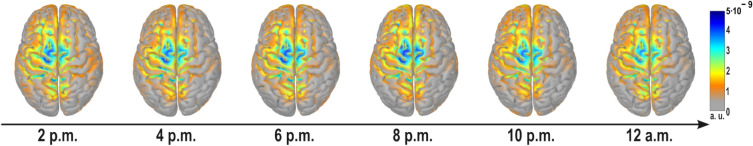
Temporal evolution of the estimated brain sources in the frequency range of 0.3 to 70 Hz at the timepoint of the motor potentials. The brain sources correspond to the grand-average over all participants and only voxels which are significantly different with respect to a baseline (parametric *t*-test, p < 0.05) are depicted. Gray areas either indicate no or no significant activity. The values depicted are unitless (a. u.). The brain images were created using Brainstorm toolbox as a MATLAB extension^41^ (Version: 3.231218, https://neuroimage.usc.edu/brainstorm/).

Through application of sLORETA for source estimation^27^, we identified brain areas exhibiting significant activity at the timepoint corresponding to the motor potential (0.067 s after movement onset) for each recording session. The reported results encompass voxels exceeding the threshold of significance with respect to a baseline (p < 0.05). The temporal dynamics in terms of activity in the broad frequency range from 0.3 to 70 Hz can be seen in Fig. 8 providing the most informative topographic representation of the brain activity. Additional topographic views showcasing brain activity within the same frequency range are available in the Supplementary Material (see Supplementary Fig. S1). In Fig. 8, for each recording session, we observed a clear activity of the primary motor cortex (M1) of the hemisphere contralateral to the movement side. Furthermore, activity was observed in the supplementary motor area (SMA) of both hemispheres, more strongly pronounced on the left side of the brain. The activity of the identified regions showed an increase from 2 p.m. to 8 p.m., followed by a subsequent decline in magnitude until 12 a.m.

**Fig. 9:**
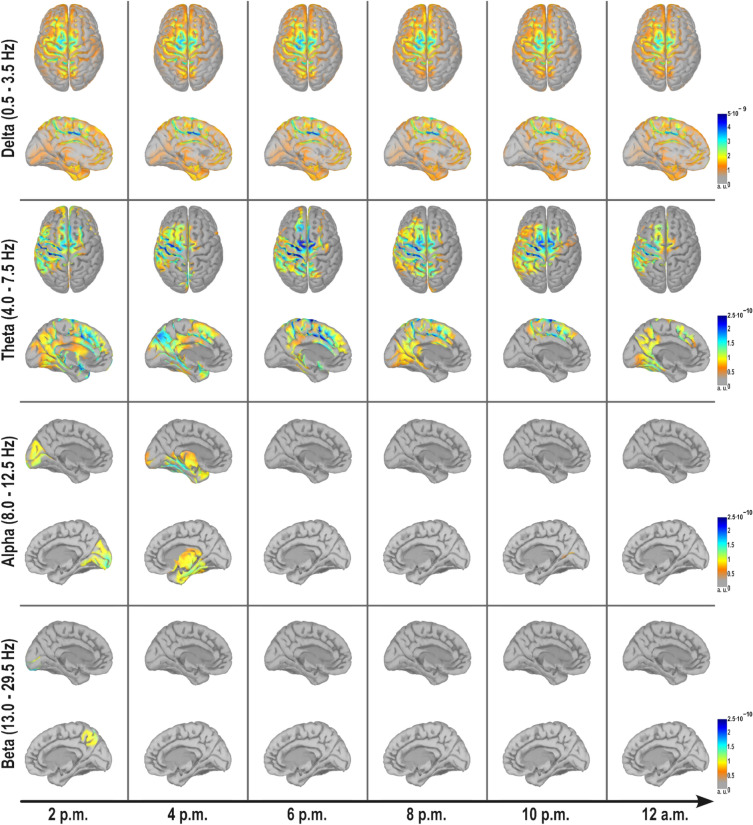
Source space analysis of changes in brain activity over time at the timepoint of the motor potential for different frequency bands and topographic views. Depicted are the grand-average sources over all participants. The visualized brain areas are those voxels which are significantly different from a baseline (parametric *t*-test, p < 0.05). Gray areas either indicate no or no significant activity. The values depicted are unitless (a. u.). For theta, alpha and beta, another range in magnitude was chosen to visualize regions of activity. The brain images were created using Brainstorm toolbox as a MATLAB extension^41^ (Version: 3.231218, https://neuroimage.usc.edu/brainstorm/).

For detailed insights into the frequency correspondence, we bandpass filtered the averaged epochs in sensor space to obtain the frequency bands in delta (0.5–3.5 Hz), theta (4.0–7.5 Hz), alpha (8.0–12.5 Hz), beta (13.0–29.5 Hz) and gamma (30.0–69.5 Hz). As previously, we employed sLORETA to estimate the brain sources and investigate the activity at the timepoint of the motor potential. Edge artifacts affecting the epochs introduced by filtering did not influence the obtained result at the timepoint of the motor potential. In Fig. 9, for each frequency band we chose two topographic views to represent the ongoing activity, with a focus on the motor area of the left hemisphere. The complete set of topographic representations for each frequency band can be found in the Supplementary Material (see Supplementary Fig. S2). Due to the absence of statistically significant activity in the gamma frequency band, we refrained from including it in the visualizations.

As shown in the broad-band activity (Fig. 8), a similar temporal pattern as in the delta band became apparent (Fig. 9). We observed sustained delta activity from 2 p.m. until 8 p.m., followed by a decline until midnight. The topographic mapping of the inside of the left hemisphere revealed that the observed activity was not confined to superficial regions but, in fact, originated from deeper brain areas proximate to the cingulate sulcus.

In contrast to the delta frequency band, theta did not reveal a consistent activation pattern of brain sources over time. For the first two measurements from 2 p.m. until 4 p.m., theta activity was present in the premotor cortex (PMC) of the contralateral hemisphere further from the longitudinal cerebral fissure and in the SMA of the ipsilateral hemisphere. Subsequently, from 6 p.m. until 10 p.m., activity shifted towards the central regions of the brain resulting in an increased activation of M1 of the left hemisphere and SMA of both hemispheres. For the measurement at 12 a.m. the same brain regions were active, albeit not as pronounced as during daytime measurements.

In the alpha frequency band, significant activation was observed only in the measurements at 2 p.m. and 4 p.m. on inner regions of both hemispheres. The activity in occipital areas at 2 p.m. transitioned towards the inner temporal lobe as well as to the thalamus and hypothalamus by 4 p.m. For the beta frequency band, significant activity was only obtained at 2 p.m. in the parietal lobe of the right hemisphere, as depicted in the sagittal view. However, for subsequent recordings, no significant brain activity was observed.

### Classification

**Fig. 10:**
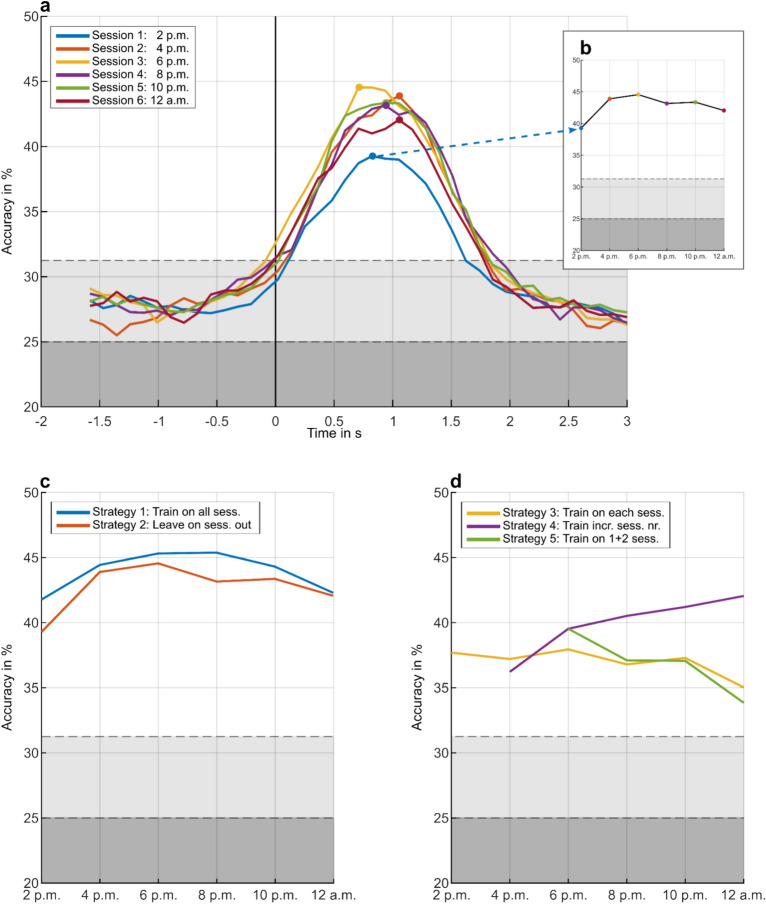
Temporal evolution of grand average accuracies for different classification strategies. (**a**) Exemplary representation of strategy 2 (leave-one-out) used to generally assess the decoding capability of data obtained at various timepoints throughout the day and night. Grand average accuracies for each session are shown by shifting a 1 s feature window along the epochs. (**b**) Change in maximum accuracy of each recording session over time for strategy 2. (**c**) Maximum accuracies over time shown for strategies 1 and 2 that give a general impression on decoding capability. (**d**) Maximum accuracies over time shown for strategies 3, 4 and 5 that indicate scenarios in which classifiers are adapted according to continuous data inflow. In all representations, the theoretical chance level was 25% for a four-gestures classification problem, shown as gray dashed line. The real chance level at 31.25% was estimated using a permutation based approach as described by Müller-Putz et al.^44^.

Figure 10a depicts the grand average accuracies for strategy 2 (leave-one-out) as an example. Six curves, each corresponding to a measurement session, illustrate the classification accuracies derived by shifting a 1 s feature window along the epochs. The classification accuracy at a given timepoint considers only current and past EEG lags meaning that classification was performed causally. Figure 10b portrays the variation in maximum classification accuracies obtained from different sessions throughout the day and night. Figure 10c,d illustrate changes in accuracies over time for the five strategies. Strategies 1 and 2 (Fig. 10c) provide a general evaluation of decoding performance using data collected at various timepoints. An increase in accuracy from 2 p.m. to 6 p.m. was observed, followed by a plateau from 6 p.m. to 8 p.m. in strategy 1, succeeded by a decline until 12 a.m. In strategy 2, accuracy decreased after 6 p.m., remained stable between 8 p.m. and 10 p.m., and then dropped at 12 a.m. Figure 10d displays the three strategies that could be applied in scenarios in which data continuously flows in. Strategy 3, which entailed training and testing on individual recording sessions, indicated a slight variation from 2 p.m. to 10 p.m., followed by a drop in accuracy at midnight. Strategy 4 that involved incorporating more data for training as time progressed, exhibited a continuous accuracy increase. In contrast, strategy 5, where the trained classifier is not updated, revealed a continuous decrease in accuracy over time. In Fig. 10, the theoretical chance level at 25% for a four-class classification task as well as the real chance level^44^ at 31.25% are indicated by the horizontal dashed lines.

## Discussion

We demonstrated that diurnal fluctuations in the EEG during movement tasks are reflected in the MRCP characteristics. Additionally, we demonstrated how temporal changes in the source space vary across frequency bands, revealing distinct patterns. Lastly, as initially hypothesized, we confirmed that changes in MRCPs over time impact motor decoding performance.

Throughout the course of the study, participants reported a progressive increase in the experience of the listed symptoms of tiredness. The duration and timing of the experiment particularly revealed an impact of exhaustion on mental resilience, specifically in terms of diminished concentration and interest. As our objective was to include participants accustomed to an earlier bedtime routine, the observed outcome is not unexpected as later measurement sessions were far past participants’ usual bedtime. In addition, symptoms related to the eyes such as burning, watering or eyelid heaviness showed an increasing trend with time compared to other symptoms. This could be attributed to the conditions during the recordings, wherein participants were positioned in front of a bright screen and instructed to refrain from blinking during trials of the gesture EEG paradigm. Consequently, this could have led to severe dryness of the eyes later in the day.

The evaluation of the VAS, targeting the level of fatigue, coincided with the findings of the TSS. Worth mentioning is that after the experimental study, participants reported that they would have answered the VAS differently in retrospect as the study turned out to be more fatiguing than they had initially expected. In terms of hunger level, the evaluation of the questionnaires met the expectations as an initial increase in hunger from 2 p.m. until the time of dinner was followed by a decrease after dinner. Regarding the answers to the PANAS questionnaires, interestingly, the positive and negative affect scores did not vary in synchrony. While the positive emotions were rated lower throughout the experiment, the negative affect scores showed an inverse correlation with hunger levels. Specifically, an increase in hunger levels until dinnertime was accompanied by a decrease in negative affect scores, indicating that participants were motivated by dinner. After dinner, fatigue manifested itself in the negative affect scores, as they exhibited an increase towards the end of the study.

Since there were no statistically significant differences observed between gestures in terms of their MRCP dynamics, it was considered appropriate to combine epochs from all gestures for subsequent analyses. We assessed temporal changes in MRCP dynamics by examining variations in the amplitude of motor and post-motor potentials. Analysis revealed that the amplitude of the motor potential showed an increase from 2 p.m. until 4 p.m. that was followed by a continuous decrease until the end of the study at 12 a.m. falling below the level of the initial measurement for all three electrodes^45^. In contrast, the evolution of the post-motor potential’s amplitude does not present the exact same pattern in all three electrodes. Nonetheless, an overall decline in amplitude between 2 p.m. and 12 a.m. could be observed. The reduction in amplitude observed for later timepoints in the day for both the motor and post-motor potential can be associated with increasing levels of fatigue and tiredness. This correlation was shown by applying LME models, which analyzed the relationship between questionnaire results on fatigue and tiredness symptoms and MRCP amplitudes. This finding has also been previously described by Dirnberger et al.^46^. Additionally, Sabeti et al.^47^ investigated the influence of mental fatigue on the P300 amplitude and documented a reduction in amplitude as an outcome of diminished cognitive attention due to mental fatigue. Previous studies have demonstrated that mental fatigue induced by prolonged sequences of motor imagery affects EEG spectral power^48^. Specifically, most significant changes were found in the frequency range of 0.1–12 Hz, which coincides with the low-frequency range of MRCPs. Additionally, researchers have investigated the influence of neuromuscular fatigue on MRCP amplitudes^49^, supporting our findings by reporting on a decrease in MRCP amplitude following a fatiguing cycling task. However, to our knowledge, this is the first study to investigate the effect of mental fatigue on MRCP characteristics, which highlights the significance and novelty of these findings.

Another possible factor contributing to a reduction in amplitude is long-time training as mentioned by Wright et al.^50,51^.

By comparing MRCP patterns between sessions, we observed that as the time interval between recordings increased, the differences in MRCP dynamics between sessions became more significant. These findings can possibly be attributed to the same factors influencing MRCPs in terms of amplitude, such as mental fatigue^46,47^, motivation^45,52^, and long-time training^50,51^.

Through source localization techniques, we demonstrated that brain activity during motor execution within the frequency range of 0.3–70 Hz and in the delta frequency band exhibits temporal variations in magnitude throughout the day. When further subdivided into narrower frequency bands, theta (4–7.5 Hz), alpha (8–12.5 Hz) and beta (13–29.5 Hz), we identified temporal fluctuations in patterns of brain sources engaged in motor execution. Both the analysis in the broad frequency range and within the delta frequency range revealed a decrease in activity of M1 and SMA contralateral to the movement side for later recordings at 10 p.m. and 12 a.m. This suggests fatigue and tiredness as potential contributing factors to the observed changes as these have been shown to affect MRCP amplitudes^46,47^. On the contrary, there were no deviations in the brain sources involved in movement execution over time, indicating consistent engagement of the same brain areas during both day- and nighttime, albeit with slight variations in activity magnitude. The consistent engagement of the same brain regions over time in the delta frequency band aligns with expectations, given that MRCPs fall within the delta frequency range.

In the theta frequency band, we noted variations in brain sources involved at the timepoint of movement execution. Similar to the delta frequency band, a decline in magnitude at 12 a.m. was observed, potentially attributed to increasing levels of fatigue and tiredness^46,47^, as reflected in the VAS assessing levels of fatigue during the gesture EEG paradigm recordings (see Supplementary Fig. S2).

Regarding the beta frequency band, significant brain activity was only evident during the initial recording session on the inside of the parietal lobe ipsilateral to the movement side. This phenomenon can be explained by beta being associated with active concentration and focused attention (for a review, see^53^). For subsequent sessions following the first, active concentration and focused attention diminish due to the consistent and repetitive nature of the experimental paradigm, which induces increasing levels of fatigue^52–54^ therefore reducing beta activity. As reported by Jap et al.^54^, a rise in fatigue additionally accounts for a decline in alpha activity in temporal brain regions, which we observed in sessions from 6 p.m. until midnight. The decrease in alpha and beta activity with time may also be associated with long-time training, as investigated by Nakano et al.^55^.

For classification, we used MRCP-based features, as indicated by previous studies. MRCPs have demonstrated to encode properties associated with movement imagination, attempt^32^ and execution^23,56^. To assess the impact of temporal changes in MRCP dynamics on classification performance, we approached the investigation from two perspectives. Initially, we incorporated samples from various timepoints of the experiment to train a decoder, aiming to establish a comprehensive evaluation of decoding capabilities for data recorded at different day and night times. This involved training on a combination of all recording sessions for strategy 1 and leaving one session out for testing in strategy 2. We observed fluctuations in decoding performance over time, characterized by a gradual increase in accuracy until 6 p.m. which was followed by a decline until midnight. This pattern could be attributed to factors such as fatigue^46^ and drowsiness, leading to a reduction in the amplitude of the MRCPs and subsequently influencing decoding capabilities. Second, through comparison of three training schemes resembling scenarios with continuous data inflow, we noted that decoders require periodic recalibration to maintain reliable and stable results at all times. Decoders trained once without the inclusion of the most recent data exhibited a decrease in classification performance over time (strategy 5). In contrast, decoders trained and tested on the same session (strategy 3) maintained almost constant performance, except for the last recording session at 12 a.m. where performance dropped. Incorporating an increasing number of training data from different recording timepoints, as shown by strategy 4, contributed to an overall performance improvement. This enhancement can be attributed to the availability of a larger dataset for training, enabling the classifier to better generalize and make more accurate predictions. Based on these findings, we conclude that variations in MRCP dynamics over time are reflected in classification capabilities.

Throughout the course of this study, participants were equipped with gel-based electrodes to conduct EEG recordings over a period of approximately 12 h. With this study, previous concerns regarding the persistence in performance of gel-based electrodes over an extensive time period can be eliminated as repeatedly performed impedances checks during each session did not show significant changes over time.

One of the main limitations of the presented study is the restricted time interval during which EEG recordings were performed. In the study, recordings only took place during the second half of a day, ignoring variations that may occur during morning periods. Nonetheless, the findings of this study are of substantial significance. We not only illustrate that MRCP patterns fluctuate throughout the day and night but also identify potential contributing factors such as fatigue and learning effects. Another aspect to consider is the role of the circadian rhythm and how biological processes affect motor-related brain activity. However, due to the nature of our study design, we were unable to directly analyze the contribution of the circadian rhythm. Furthermore, given the involvement of multiple factors, disentangling the effects of the circadian rhythm from those of training and fatigue is not a straightforward procedure. Future investigations into temporal variations in MRCP dynamics could incorporate a 24-h time period during which recordings are made in order to explore the stability of the MRCP dynamics from 1 day to another, hence exploring the influence of the circadian rhythm on EEG during movement tasks. Another limitation to mention is that we did not apply a closed-loop BCI. We are aware that the feedback might alter MRCP characteristics and user engagement^57^. Also, it might happen that when individuals with motor impairments get involved, MRCP shape or specific components^18,58^ may be altered. However, studies on people with spinal cord injury did not show such changes yet^59^. Future research should incorporate closed-loop BCI setups and target the specific MRCP characteristics of potential BCI users to ensure the generalizability and applicability of the findings.

## Conclusion

In this study we showed for the first time that variations in the EEG during movement tasks persist across both day and night periods. We demonstrated temporal variations in MRCP dynamics within both the sensor and source space, shedding light on their impact on decoding capabilities. Based on these observations, we draw the conclusion that achieving consistent and robust decoding of brain signals at all times necessitates the deployment of adaptive decoders.

These findings will contribute to the implementation of adaptive decoders to be integrated in the implantable BCI system in order to build a reliable and satisfactory BCI technology that allows LIS patients to communicate without the help of their caregiver, hence enhancing their autonomy and independence.

### Supplementary Information

Supplementary Figures.

## Data Availability

The datasets used and analysed during the current study available from the corresponding author on reasonable request.
