# Supplementary material for: Chrono-EEG dynamics influencing hand gesture decoding: a 10-hour study
Source: Sci Rep. 2024 Aug 30;14:20247. doi: 10.1038/s41598-024-70609-x (PMC11364647; doi:10.1038/s41598-024-70609-x)
Supplement: Supplementary file 1 — Supplementary Figures. [file 41598_2024_70609_MOESM1_ESM.pdf]

Supplementary Material:

Chrono-EEG dynamics influencing hand gesture decoding: A 10-hour study

Johanna Egger[1], Kyriaki Kostoglou[1] and Gernot R. Müller-Putz[1,2]

1 Institute of Neural Engineering, Graz University of Technology, Graz, Austria

2 BioTechMed Graz, Austria

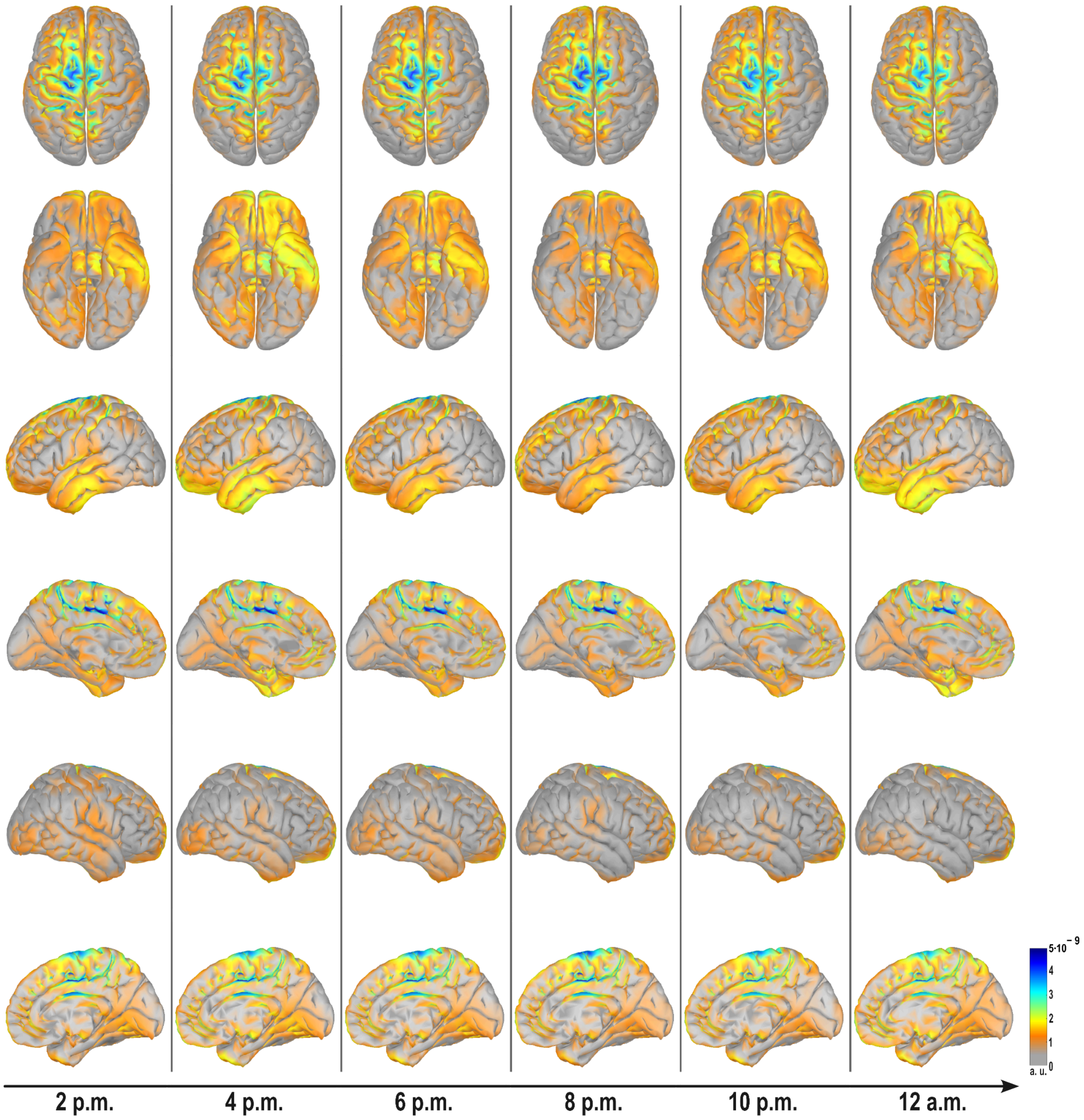

Supplementary Figure S1: Temporal dynamics of estimated brain sources at the time point corresponding to the motor potential within the frequency range of 0.3 to 70 Hz, illustrated from various topographic perspectives. The average across participants is shown whereas only voxels exhibiting statistical significance with regard to a baseline (parametric t-test, $p < 0.05$) are visualized. Areas with highest activity are highlighted in blue which corresponds to the primary motor cortex of the left hemisphere and supplementary motor area of both hemispheres. Regions devoid of significant activity are delineated in gray. Depicted values are unitless (u. a.). The brain images were created using Brainstorm toolbox as a MATLAB extension [1] (Version: 3.231218, https://neuroimage.usc.edu/brainstorm/).

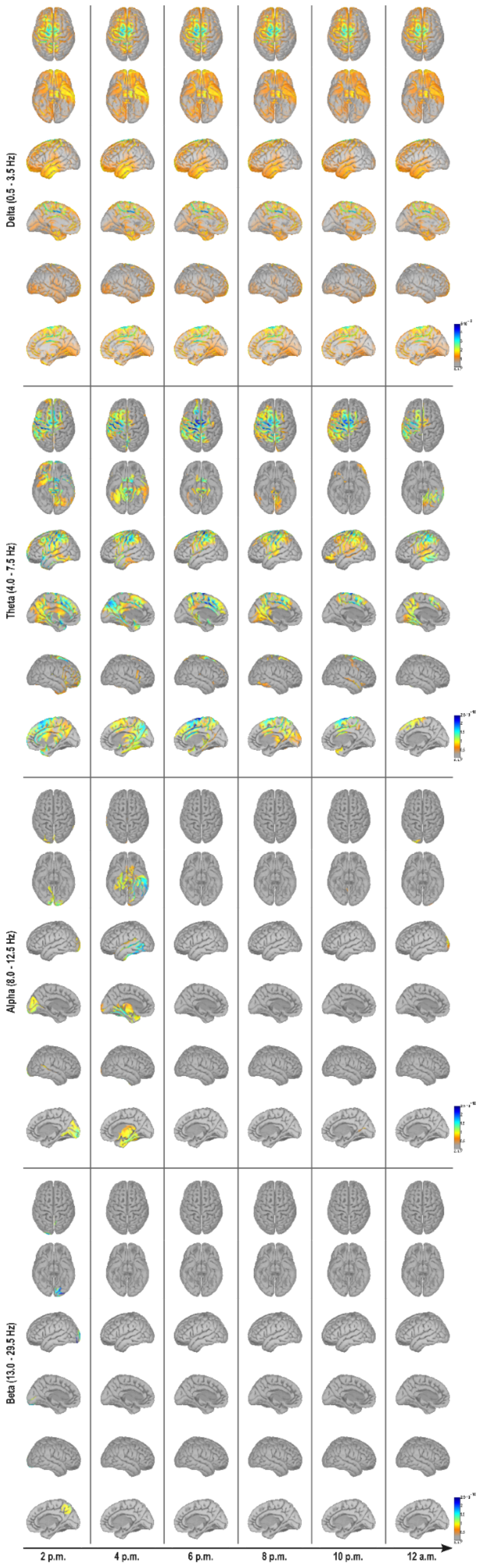

Supplementary Figure S2: Changes in brain activity projected to the source space over time shown for specific frequency bands and topographic representations. In gray, brain areas with no significant or lack of activity are shown whereas blue areas mark regions with highest activity, values are unitless (u. a.). The sources depicted are the grand-average over all participants showing only voxels with statistical significance in comparison to a baseline (parametric t-test, $p < 0.05$). The brain images were created using Brainstorm toolbox as a MATLAB extension [1] (Version: 3.231218, https://neuroimage.usc.edu/brainstorm/).

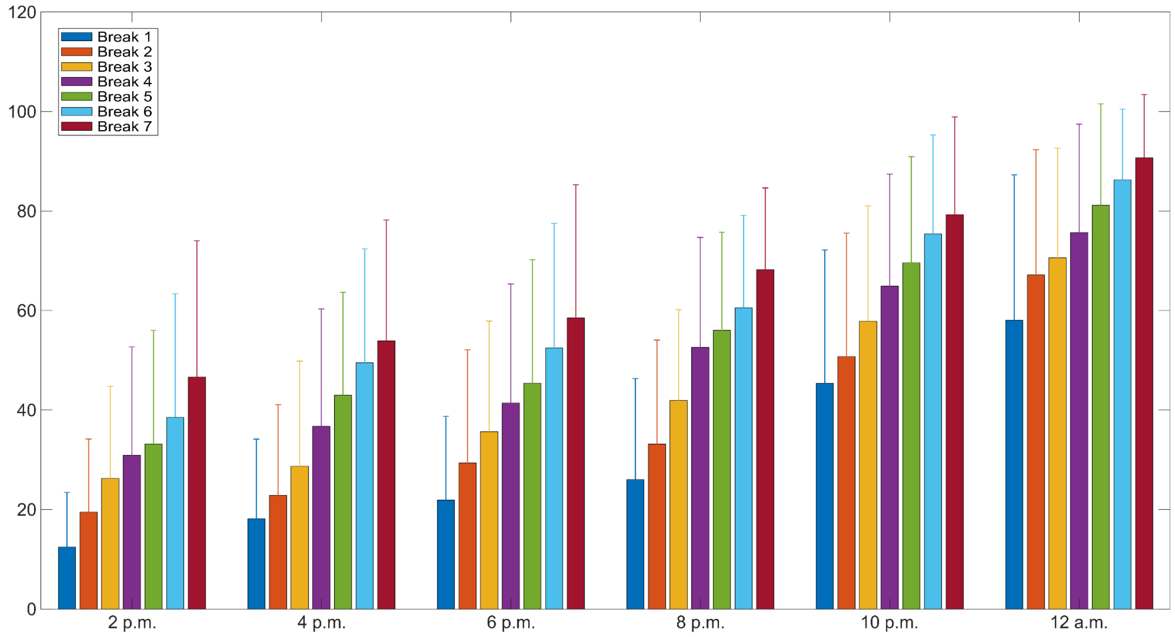

Supplementary Figure S3: Evaluation of the visual analogue scale to assess the level of fatigue for each one of the seven breaks during the gesture EEG paradigm of each measurement session. Participants were instructed to state their level of fatigue after each 5 min block of the EEG recording of the gesture paradigm for every session. Values range from 0 (not at all fatigued) to 100 (extremely fatigued). The representation shows the mean across participants as bar plots including error bars.

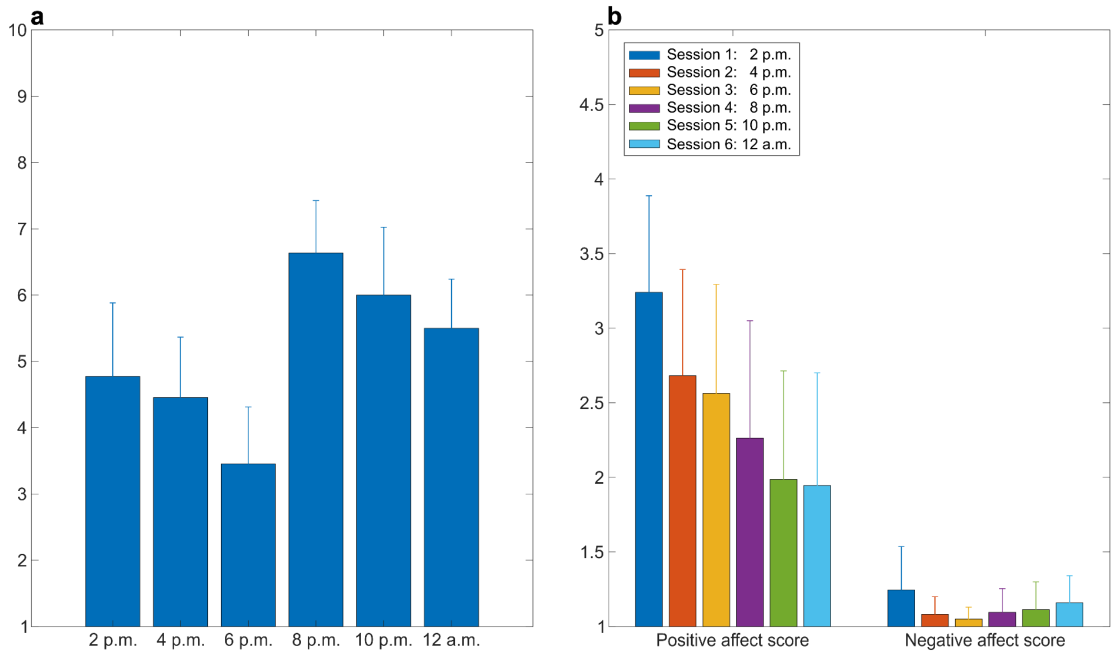

Supplementary Figure S4: Evaluation of the (a) Hunger Level Scale and (b) PANAS of all participants for each measurement session. (a) shows the hunger level ratings which range from 1 (starvation) to 10 (full, uncomfortable feeling of the stomach) for each session. An increase in hunger can be seen from 2 p.m. until 6 p.m., at 7 p.m. dinner was served which is marked by the abrupt decrease in hunger at 8 p.m. From 8 p.m. until 12 a.m. a slight increase in hunger can be observed. (b) indicates the positive (left) and negative (right) affect score for each session. Ratings ranged from 1 (low) to 5 (high). Obvious is the decline in positive affect score from 2 p.m. until 12 a.m. For the negative affect score, we determine a decrease from 2 p.m. until 6 p.m. which is followed by a gradual increase until 12 a.m. after dinner was served.

References

1. Tadel F, Baillet S, Mosher JC, Pantazis D, Leahy RM. Brainstorm: a user-friendly application for MEG/EEG analysis. Comput Intell Neurosci. 2011 Apr 13;2011:879716.
